# Comparison of the Efficacy of Corticosteroids With Enzymatic Agents in the Postoperative Sequelae for Lower Third Molar Surgery: A Prospective Study

**DOI:** 10.7759/cureus.55397

**Published:** 2024-03-02

**Authors:** Goutham Vijayakumar, Gidean A Sundaram, Santhosh P Kumar, Murugesan Krishnan, Vinod K Krishna, Saravanan Lakshmanan

**Affiliations:** 1 Oral and Maxillofacial Surgery, Saveetha Dental College and Hospitals, Saveetha Institute of Medical and Technical Sciences, Saveetha University, Chennai, IND

**Keywords:** postoperative swelling, mandibular third molar surgery, enzymatic agents, papain, rutoside, bromelain, chymotrypsin, trypsin, serratiopeptidase, prednisolone

## Abstract

Introduction

The presence of impacted third molars is a prevalent problem associated with varying degrees of difficulty in extraction and potential consequences, including pain, swelling, and trismus. According to studies, enzymatic combinations, such as bromelain, rutoside, trypsin, and serratiopeptidase, are known to have a very promising role in reducing inflammation and promoting wound healing. This study compared natural enzymatic agents with corticosteroids for postoperative pain, swelling, and trismus in the impacted lower third molar surgery.

Objectives

The present study aimed to compare the efficacy of prednisolone, a combination of trypsin, chymotrypsin, bromelain, rutoside, and papain, and serratiopeptidase in the postoperative sequelae after surgical extraction of impacted mandibular third molars. The primary objective was to assess the difference in swelling between the three groups. The secondary objectives were to assess the difference in postoperative pain and trismus between the three groups.

Materials and methods

A total of 150 patients who presented to the department of oral and maxillofacial surgery for surgical removal of an impacted mandibular third molar with a moderately difficult score of 5-7 in the Pederson difficulty index were chosen for a prospective study. Patients were categorized into three groups based on the postoperative drug prescribed. In group 1, prednisolone 10 mg was prescribed; in group 2, a combination of trypsin, chymotrypsin, bromelain, rutoside, and papain was prescribed; and in group 3, serratiopeptidase 15 mg was prescribed. All patients were prescribed a combination drug of aceclofenac 100 mg and paracetamol 325 mg twice daily as a standard analgesic. Swelling, pain, and trismus in each patient were recorded preoperatively and at postoperative day one and day seven. The Friedman test was employed to evaluate the variation in pain levels within the groups over time, while the Kruskal-Wallis test was utilized to investigate the disparity in pain levels between the groups. The difference in swelling and trismus within the groups across the timeline was measured by repeated measures analysis of variance (ANOVA), and the difference in swelling and trismus between the groups was measured by one-way ANOVA. A p-value below 0.05 was deemed to be statistically significant.

Results

Group 1 showed less swelling, pain, and trismus on both postoperative day one and day seven compared to group 2 and group 3, which was a statistically significant difference (P < 0.05). It was found that swelling, pain, and trismus measurements in postoperative day one and day seven in group 2 were comparatively less than in group 3. Neither group demonstrated any side effects or other complications during the follow-up period.

Conclusion

It can be concluded that the use of prednisolone postoperatively following surgical removal of the mandibular third molar provided better relief with regard to pain, trismus, and swelling compared to the enzymatic agents. Among enzymatic agents, a combination of trypsin, chymotrypsin, bromelain, rutoside, and papain was better in reducing pain, trismus, and swelling than serratiopeptidase drug.

## Introduction

One of the most popular dento-alveolar procedures is the extraction of impacted lower third molars, which is accompanied by variable levels of patient discomfort. Pain, swelling, and trismus are reduced when nearby soft tissues sustain minimal damage and when wounds are closed properly. It follows that pharmacologic approaches aimed at reducing the clinical signs of surgical trauma should naturally focus on preventing the synthesis of acute inflammatory mediators or reducing their impact [1]. After surgery, enzymes have been shown to have anti-inflammatory, antithrombotic, and fibrinolytic properties in vitro and in vivo. This helps to reduce the risk of complications following surgery. Since impacted third-molar extractions are typically performed on teenagers and young adults, it is generally accepted that elderly patients have a higher chance of complications following third-molar surgery. It is widely acknowledged that men and women react to pain stimuli and perceive them differently [2]. Furthermore, there are biological variations between men and women that may influence how they react to medications. Therefore, it makes sense that pharmacologic approaches to reduce the clinical signs of surgical injury would focus on preventing the synthesis of acute inflammation mediators or reducing their effects [3,4].

The present study aimed to compare the efficacy of prednisolone, a combination of trypsin, chymotrypsin, bromelain, rutoside, and papain, and serratiopeptidase in the postoperative sequelae after surgical extraction of impacted mandibular third molars. The primary objective was to assess the difference in swelling between the three groups. The secondary objectives were to assess the difference in postoperative pain and trismus between the three groups.

## Materials and methods

Study design and setting

This prospective comparative study was conducted on 150 patients who reported to the Department of Oral and Maxillofacial Surgery at Saveetha Dental College, Chennai from March 2023 to September 2023 for surgical removal of an impacted mandibular third molar with a moderately difficult score of 5-7 in the Pederson difficulty index (1988). Following a thorough history-taking, patients underwent a clinical examination and were informed about the procedure, potential complications, and the study's follow-up period. This study was approved by the Institutional Human Ethical Committee (IHEC/SDC/OMFS-2205/22/333) and it was performed after obtaining written informed consent from the study participants.

Inclusion criteria

The study comprised patients who were categorized by the American Society of Anesthesiologists (ASA) physical status classification system as ASA I or ASA II. The participants were individuals requiring the surgical extraction of horizontally impacted mandibular third molars with class 2 and position B with no symptoms or swelling one-week pre-surgery. The patients were also not on any antibiotics or anti-inflammatory medications one week before the surgery.

Exclusion criteria

Patients with ASA classification III or higher, pre-existing infections, diabetes mellitus, or uncontrolled hypertension; women who were pregnant, lactating, or using oral contraceptives; patients with drug allergies; and smokers were excluded from the study.

Study population

The 150 patients were split into three groups of 50 each. There were a total of 82 males and 68 females, with a mean age of 27 ± 4.3 years. Baseline data were obtained by recording the inter-incisal opening, pain scores, and facial swelling measurements before surgery. All third molar extractions were performed on patients by the same clinician and a review was done by a different examiner who was blinded to the type of postoperative drug used. Postoperative instructions for surgical extraction were provided. Amoxicillin 500 mg was given to each patient three times a day for three days. All patients were prescribed a combination of aceclofenac 100 mg and paracetamol 325 mg twice daily as a standard analgesic. Enzymatic drugs or corticosteroids were prescribed along with antibiotics and analgesics. Patients were categorized into three groups based on the following postoperative drug administered: group 1 - tablet prednisolone 10 mg twice daily after food for three days; group 2 - tablet combination of trypsin, chymotrypsin, bromelain, rutoside, and papain twice daily after food for three days; and group 3 - tablet serratiopeptidase 15 mg twice daily after food for three days. Subsequent assessments were conducted on the first and seventh postoperative days and were compared to the baseline.

Assessment

**Figure 1:**
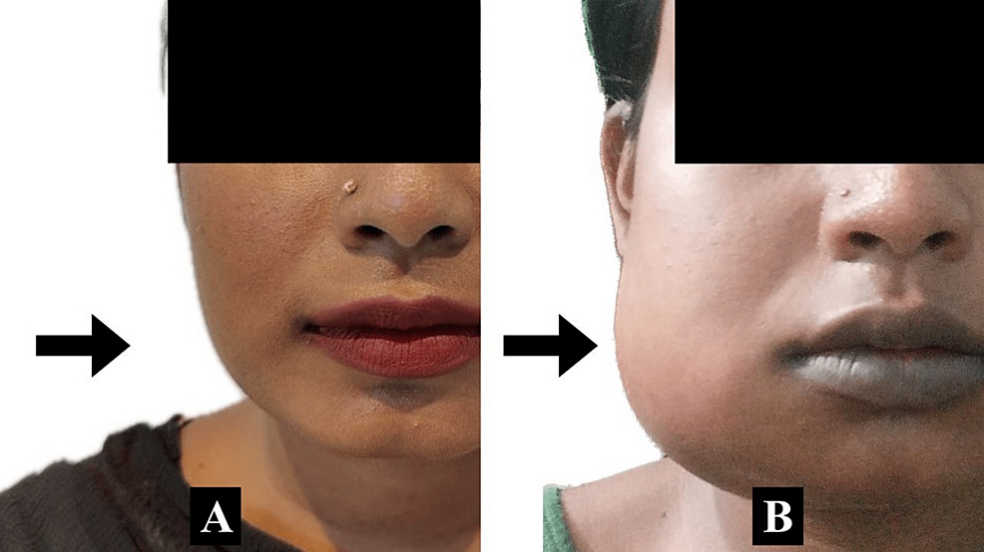
Preoperative and postoperative swelling following surgical removal of impacted mandibular right third molar in a group 3 patient. (A) Preoperative; (B) postoperative day one.

**Figure 2:**
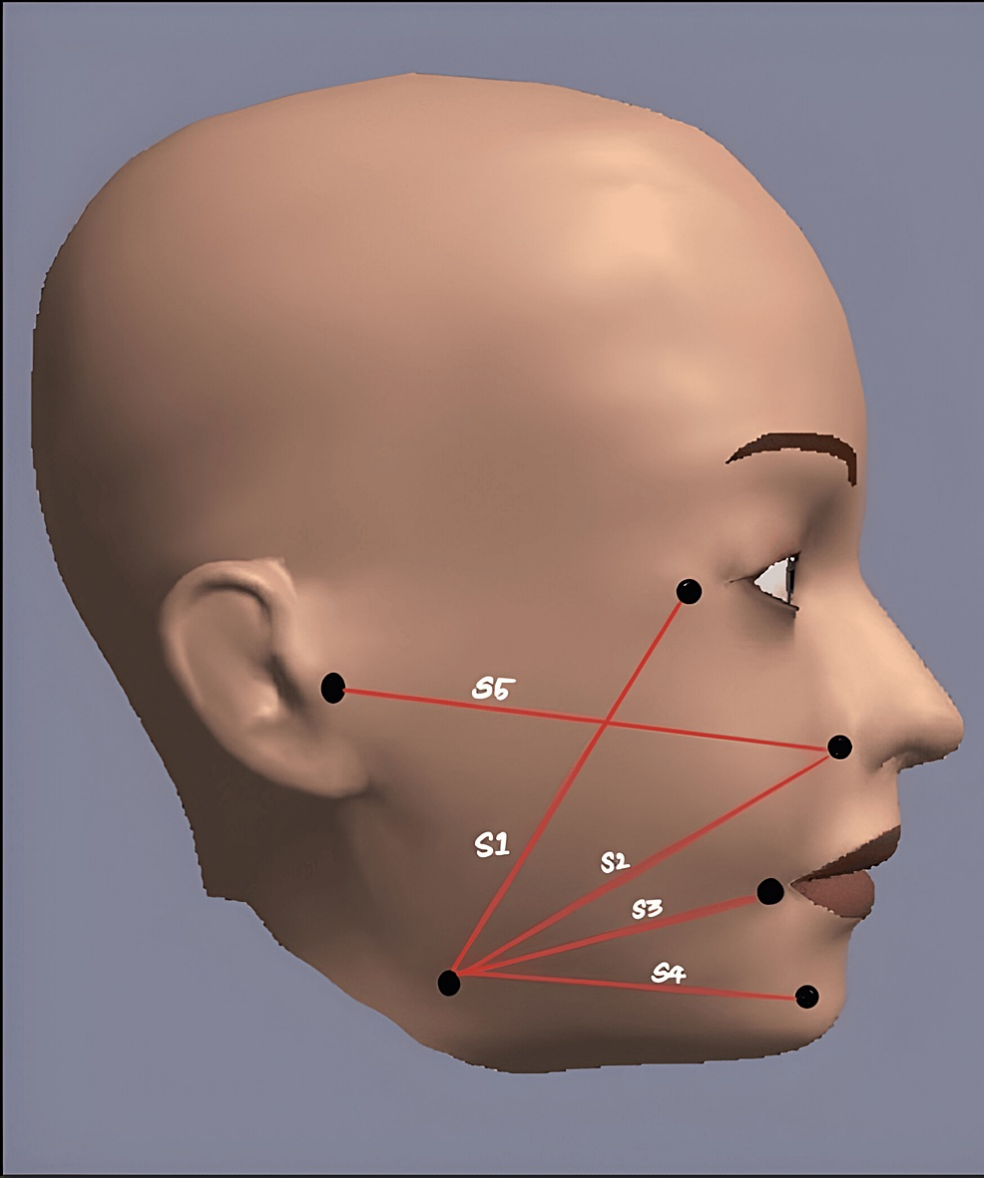
Facial markings taken to measure the possible swelling areas. S1: from the lateral canthus of the eye to the angle of the mandible; S2: from ala of the nose to the angle of the mandible; S3: from the corner of the mouth to the angle of the mandible; S4: from the menton to the angle of the mandible; S5: from the ala of the nose to the tragus of the ear. Figure credits: Figure created by the first author with Sketchbook software 6.0.5 (iOS).

The outcome parameters assessed were swelling, pain, and trismus. The primary outcome was swelling, and the secondary outcomes measured were pain and trismus. Swelling at preoperative, postoperative day one, and day seven in each patient was recorded. Figure 1 shows a comparison between preoperative and postoperative swelling (day one) in a group 3 patient. The swelling was measured using a five-line tape measurement as depicted in Figure 2. Pain was assessed by a Visual Analog Scale ranging from 0 to 10 with 0 indicating no pain, 5 indicating moderate pain, and 10 indicating worst pain. The evaluation of trismus involved assessing the maximum mouth opening by measuring the inter-incisal distance.

Statistical analysis

The data were analyzed using SPSS for Windows, version 23.0 (IBM Corp., Armonk, NY). The normality of the data was assessed using the Shapiro-Wilk test. The Friedman test was employed to evaluate the variation in pain levels within the groups over time, while the Kruskal-Wallis test was utilized to investigate the disparity in pain levels between the groups. The difference in swelling and trismus within the groups across the timeline was measured by repeated measures analysis of variance (ANOVA), and the difference in swelling and trismus between the groups was measured by one-way ANOVA. A p-value below 0.05 was deemed to be statistically significant.

## Results

The study population consisted of 150 participants who underwent surgical removal of impacted mandibular third molars. The population was divided into three groups, with 50 participants in each group. There were a total of 82 males and 68 females, with a mean age of 27 ± 4.3 years. The effect of the administration of oral prednisolone with the other two groups of enzymatic agents on postoperative symptoms in patients undergoing impacted lower third molar extractions was assessed.

The mean swelling scores within the groups increased from the preoperative day to the first postoperative day and later reduced on the seventh postoperative day in all groups. The results were statistically significant (p < 0.05), with the lowest scores observed in group 1, followed by group 2 and group 3. The mean swelling scores between the groups before the surgery did not show any statistically significant difference (p = 0.846). The mean swelling scores differed significantly between the groups on the first postoperative day, with group 1 showing the least swelling and group 3 exhibiting the most swelling (p = 0.023). The mean swelling ratings on the seventh postoperative day differed significantly between the groups. Group 1 had the least swelling, whereas group 3 had the most swelling (p = 0.047) (Table 1).

**Table 1:** Mean swelling in millimeters among the study groups. Data are represented as mean ± standard deviation (SD). * Statistically significant (p < 0.05). Within-group comparison - repeated measures analysis of variance test. Between-group comparison: one-way analysis of variance test.

Mean swelling score	Group 1	Group 2	Group 3	P-value
Preoperative	108.71±4.26	109.66±7.13	111.62±2.08	0.846
Postoperative day 1	112.45±4.42	117.23±5.53	127.54±1.48	0.023*
Postoperative day 7	109.59±2.63	112.25±2.51	118.76±2.55	0.047*
P-value	0.002*	0.001*	0.042*	

The mean pain scores within the groups decreased from the preoperative day to the seventh postoperative day in all groups, and the results were statistically significant (p < 0.05). The lowest scores were observed in group 1, followed by group 2 and group 3. The difference in mean pain scores between the groups preoperatively was not statistically significant (p = 0.748). However, the difference in mean pain scores between the groups on the first postoperative day was statistically significant, with the least pain exhibited in group 1 and the maximum pain in group 3 (p = 0.012). The difference in mean pain scores between the groups on the seventh postoperative day was also statistically significant, with the least pain exhibited in group 1 and maximum pain in group 3 (p = 0.047) (Table 2).

**Table 2:** Mean pain score in the Visual Analog Scale among the study groups. Data are represented as mean ± standard deviation (SD). * Statistically significant (p < 0.05). Within-group comparison - Friedman test. Between groups comparison - Kruskal-Wallis test.

Mean pain score	Group 1	Group 2	Group 3	P-value
Preoperative	8.55±0.82	7.18±1.4	7.64±1.69	0.748
Postoperative day 1	4.64±1.5	6.09±1.44	7.36±1.69	0.012*
Postoperative day 7	1.09±0.94	1.55±1.03	2.27±1.68	0.047*
P-value	0.036*	0.029*	0.032*	

The mean trismus scores within the groups decreased from the preoperative day to the seventh postoperative day in group 1, and the results were statistically significant (p < 0.05). The mean trismus scores within the groups decreased from the preoperative day to the first postoperative day and later increased in the seventh postoperative day in groups 2 and 3, and the results were statistically significant (p < 0.05). Maximal mouth opening (less trismus) was observed in group 1, followed by group 2 and group 3. The difference in mean trismus scores between the groups preoperatively was not statistically significant (p = 0.423). However, the difference in mean trismus scores between the groups on the first postoperative day was statistically significant, with the least trismus (maximal mouth opening) exhibited in group 1 and the maximum trismus in group 3 (p = 0.014). The difference in mean trismus scores between the groups on the seventh postoperative day was also statistically significant, with the least trismus (maximal mouth opening) exhibited in group 1 and maximum trismus in group 3 (p = 0.008) (Table 3).

**Table 3:** Mean trismus in millimeters among the study groups. Data are represented as mean ± standard deviation (SD). * Statistically significant (p < 0.05). Within group comparison - repeated measures analysis of variance test. Between groups comparison - one-way analysis of variance test.

Mean trismus score	Group 1	Group 2	Group 3	P-value
Preoperative	42±4.79	45.18±5.01	38.63±5.81	0.423
Postoperative day 1	41.27±6.48	33.72±6.54	25.36±6.2	0.014*
Postoperative day 7	40.18±6.61	39.18±6.61	31.18±6.61	0.008*
P-value	0.017*	0.028*	0.003*	

## Discussion

Most often, patients are terrified of the pain involved in having their wisdom teeth extracted. Analgesics can be used to manage postoperative pain by bringing it down to a manageable level. Corticosteroids have primarily been used to reduce postoperative swelling and restricted mouth opening, but when used at the proper dosage and timing during the procedure, they can also have analgesic effects [5]. By reducing the production of vasoactive substances and cytokines, corticosteroids act by suppressing every stage of the initial inflammatory response, which lowers cellular permeability and capillary dilatation. Moreover, corticosteroids suppress prostaglandin synthesis, which has an analgesic effect [5,6].

Perioperative administration of corticosteroids produced a mild to moderate reduction in edema and an improvement in the range of jaw motion after the third molar removal. Treatment with corticosteroids was associated with significantly less edema during and late assessments and less trismus (measured as improved jaw movement) at early assessment and late assessment than control treatments. Steroid medication has proved to be well suited to the treatment of postoperative pain, trismus, and swelling after dental surgical procedures [7]. Adjunctive analgesics include steroids. Although their use is primarily not in pain management, they have demonstrated analgesic properties in certain painful situations. These medications are called secondary analgesics, co-analgesics, auxiliary analgesics, and non-indication drugs, all of which have different mechanisms of action [6,7]. As a result, the majority of steroids only function as adjuvants to prevent oral soft tissue edema and inflammation, which aggravate pain. They can be taken with analgesics to lessen pain and other inflammatory symptoms.

The recommended medications for third-molar intervention that can successfully reduce pain following impacted mandibular third-molar surgery are corticosteroids like methylprednisolone and dexamethasone. The best-preferred routes of drug administration for reducing pain are intramuscular and intravenous routes. The preoperative stage is the ideal time to administer an injection to get the most pain-relieving effects from corticosteroids [8]. Because of their indirect analgesic effects, corticosteroids can have analgesic qualities but are not classified as analgesic medications. They can be taken in addition to analgesics to lessen pain and other inflammatory symptoms. A single dose of prednisolone controlled the trismus, pain, and facial swelling following the surgical removal of impacted third molars. The postoperative administration of 30 mg of prednisolone is effective in modulating the intensity of both clinical parameters, i.e., pain and swelling, according to a randomized, single-dose clinical study, which elicited surgical removal of impacted third molars [9]. When third molar surgeries are performed, corticosteroids are frequently used to manage postoperative inflammation. The role of corticosteroids in the management of postoperative pain relies on the analgesic and anti-inflammatory properties of corticosteroids, which would possibly enable the patient to relieve postoperative distress [10].

The enzymes serratiopeptidase, trypsin, chymotrypsin, bromelain, rutoside, and papain have a unique mechanism of action in the management of postoperative pain [11]. When an injury occurs, the periapical tissues release inflammatory mediators like prostaglandins, leukotrienes, bradykinins, and other neuropeptides, which sensitize the pain fibers. Furthermore, tissue edema and elevated interstitial tissue pressure can be brought on by vasodilation, increased vascular permeability, and the inflammatory cells' chemotaxis [12]. Trypsin and chymotrypsin from pigs and cows are used to make the proteolytic enzyme supplement known as the trypsin-chymotrypsin complex. This enzyme complex acts as an anti-inflammatory, analgesic, anti-edematous, fibrinolytic, and anti-infective agent once it is taken orally and absorbed into the bloodstream. Consequently, this facilitates a quicker resolution of inflammatory symptoms and offers superior analgesia in contrast to other proteolytic enzymes [13].

A combination of enzymes called bromelain is extracted from the stem of the *Ananas comosus* pineapple plant. Several enzymes exhibiting proteolytic activity are among its primary active ingredients [14,15]. While bromelain's proteolytic fraction plays a significant role, other factors also contribute to many of its positive effects. Oral administration of bromelain has been shown to have anti-inflammatory and analgesic properties in several human studies, in addition to evidence from animal studies [16,17]. Bradykinin's inhibition of prostaglandin synthesis and its blocking are the mechanisms by which bromelain exerts its anti-inflammatory effects. Due to its apparent anti-edemic, anti-inflammatory, and anticoagulation properties that can aid in postoperative healing and the fact that it is most effective when taken orally, bromelain has drawn special attention from the plastic surgery community [17].

The combination of non-steroidal anti-inflammatory drugs plays an important role in the surgical removal of impacted teeth. The proteolytic enzyme bromelain is helpful in edematous and inflammatory areas. It works by blocking the pro-inflammatory metabolites that fuel inflammation [18]. Another proteolytic enzyme produced when trypsinogen is activated is trypsin [19]. It works by enhancing the humoral response and inhibiting the spread of pathogens. Rutoside acts by blocking macrophage mediators of inflammation and arthritis. It is an anti-inflammatory antioxidant that inhibits the damaging free radicals produced during inflammation [20]. The mechanism of action of papain and bromelain involves breaking down proteins in the body. They are very similar to pepsin, which is a naturally occurring enzyme that plays a large role in digestion by breaking down proteins in food [18].

Limitations of the study

This study aimed to compare the gold standard corticosteroids with available enzymatic agents in reducing swelling, pain, and trismus after third molar surgery. This study compared only two available enzymatic combinations, but other enzymatic agents like curcumin are also available, which may be evaluated in future research with a larger sample size.

## Conclusions

The use of prednisolone after the surgical removal of the impacted mandibular third molar provided the best relief with regard to swelling, pain, and trismus compared to other enzymatic agents. Among enzymatic agents, a combination of enzymes like trypsin, chymotrypsin, bromelain, rutoside, and papain was found to be better at reducing swelling, pain, and trismus than serratiopeptidase as a postoperative drug in lower third molar surgery.
